# Use of whole genome sequencing in surveillance for antimicrobial-resistant *Shigella sonnei* infections acquired from domestic and international sources

**DOI:** 10.1099/mgen.0.000270

**Published:** 2019-05-17

**Authors:** Rebecca L. Abelman, Nkuchia M. M'ikanatha, Hillary M. Figler, Edward G. Dudley

**Affiliations:** 1 Department of Food Science, The Pennsylvania State University, University Park, Pennsylvania, USA; 2 Pennsylvania Department of Health, Harrisburg, Pennsylvania, USA; 3 Huck Institutes of the Life Sciences, The Pennsylvania State University, University Park, Pennsylvania, USA; 4 *E. coli* Reference Center, The Pennsylvania State University, University Park, Pennsylvania, USA

**Keywords:** *Shigella sonnei*, antibiotic resistance, microbial phylogenetics

## Abstract

*Shigella* species are a major cause of gastroenteritis worldwide, and *Shigella sonnei* is the most common species isolated within the United States. Previous surveillance work in Pennsylvania documented increased antimicrobial resistance (AMR) in *S. sonnei* associated with reported illnesses. The present study examined a subset of these isolates by whole genome sequencing (WGS) to determine the relationship between domestic and international isolates, to identify genes that may be useful for identifying specific Global Lineages of *S. sonnei* and to test the accuracy of WGS for predicting AMR phenotype. A collection of 22 antimicrobial-resistant isolates from patients infected within the United States or while travelling internationally between 2009 and 2014 was chosen for WGS. Phylogenetic analysis revealed both international and domestic isolates were one of two previously defined Global Lineages of *S. sonnei*, designated Lineage II and Lineage III. Twelve of 17 alleles tested distinguish these two lineages. Lastly, genome analysis was used to identify AMR determinants. Genotypic analysis was concordant with phenotypic resistance for six of eight antibiotic classes. For aminoglycosides and trimethoprim, resistance genes were identified in two and three phenotypically sensitive isolates, respectively. This article contains data hosted by Microreact.

## Data Summary

1. Isolates sequenced in this study from the Pennsylvania Department of Health collection were deposited in the National Center for Biotechnology Information (NCBI), under BioProject Number PRJNA273284 (https://www.ncbi.nlm.nih.gov/bioproject/?term=PRJNA273284). Corresponding accession numbers can be found in Table S1 (available in the online version of this article).
2. A list of other sequences (and references, if available) utilized in this study can be found in Table S2. All sequences were downloaded from the NCBI Sequence Read Archive.
3. The complete annotated genome sequence of *Shigella sonnei* Ss046 was downloaded from NCBI (https://www.ncbi.nlm.nih.gov/nuccore/CP000038.1).

**Significance as a BioResource to the community**

Whole genome sequencing has become an integral part of characterizing *Shigella* spp., however limited studies have focused on *S. sonnei* isolated in North America. This study demonstrated that a collection of *S. sonnei* isolates from the state of Pennsylvania segmented phylogenetically into previously described Global Lineages II and III. These findings add to the only other similar study in the United States, which identified primarily Lineage III isolates in California. Comparison of AMR predicted by genotype with *in vitro* susceptibility data provides additional information concerning the feasibility of predicting *S. sonnei* resistance phenotypes using only whole genome sequence data. Lastly, comparison of *S. sonnei* sequenced here with publicly available genomes identified SNPs that may be useful for identifying Lineage II and III isolates, and suggests that a previously developed SNP-based tool for Lineage classification should be revisited to consider the more recently described Lineage V.

## Introduction

*Shigella* species are agents of bacillary gastrointestinal illness responsible for an estimated 80–165 million cases worldwide [1, 2]. Additionally, *Shigella* is the fourth most common cause of bacterial foodborne illnesses in the USA [3]. There are four recognized species of *Shigella*; in the USA and other developed countries, *Shigella sonnei* accounts for over 80 % of all shigellosis infections [4, 5]. *S. sonnei* is increasingly found in areas of the world undergoing industrialization [6].

Genomic analysis of *S. sonnei* isolates primarily from Asia, Europe, Africa, and South and Central America defined five lineages [7, 8]. Lineages I and IV were primarily found in Europe, while isolates of Lineage III appear to be the most widespread [8]. These studies, however, did not include any *S. sonnei* from the USA. A recent study in California concluded that Lineage III isolates were responsible for outbreaks occurring between 2014 and 2015 in the San Diego, San Joaquin and San Francisco areas [9]. Whether this lineage dominates in other regions of the USA is unknown.

As with several other enteric pathogens, antimicrobial resistance (AMR) in *S. sonnei* is of concern. In a recent report by the National Antimicrobial Resistance Monitoring System (NARMS) in 2015, 2.5 % of all *Shigella* isolated from humans in the USA were resistant to ciprofloxacin and 9.8 % were resistant to azithromycin, the first-line antibiotics typically used to treat shigellosis [10]. Additionally, the report showed that over 41 % of *Shigella* isolated were resistant to three or more classes of antibiotics. Several studies have shown that whole genome sequencing (WGS) is an accurate predictor of *in vitro* antimicrobial resistance. For example, with *Escherichia coli* and other Gram-negative bacteria, greater than 97 % correlation has been found between the genomic and phenotypic methods [11–14]. To our knowledge, correlation of phenotypic *S. sonnei* antimicrobial susceptibility results to genomic antimicrobial analysis has only been done on isolates from the UK and California [9, 15]. Additional research on other collections would be useful to gauge the effectiveness of AMR detection via genome analysis when compared to traditional *in vitro* testing, specifically on a wider range of *S. sonnei* isolates.

Previously, the Pennsylvania Department of Health characterized AMR patterns of *Shigella* isolates associated with reported illnesses during 2006–2014 [16]. This study reported increased resistance to clinically important drugs (e.g. azithromycin or ciprofloxacin) in *S. sonnei*. To increase our understanding of the genetic lineages of *S. sonnei* and the accuracy of predicting AMR patterns using WGS data, we selected 22 isolates from the original study, focusing on domestic and internationally acquired isolates that were previously determined to be resistant to multiple classes of antibiotics.

## 

**Table 1.** *S.sonnei* isolates sequenced in this study

PSU-ID*	**Travel history†**	**Year of isolation**	**AMR profile‡**
SS-2	None	2011	STR, AMP, AMC, FOX, TET
SS-3	None	2013	GEN, STR, SXT, SMX, TET, AZM
SS-4	None	2013	STR, SXT, SMX, TET
SS-5	None	2014	STR, AMP, SXT, SMX, TET, AZM
SS-21	India	2009	STR, SXT, NAL, SMX, TET
SS-23	Nepal	2009	STR, SXT, CIP, NAL, SMX, TET
SS-24	India	2010	STR, SXT, CIP, NAL, SMX, TET
SS-26	Jamaica	2010	STR, SXT, NAL, SMX, TET
SS-27	None	2010	STR, AMP, SXT, SMX, TET
SS-28	None	2011	STR, AMP, AMC, FOX
SS-29	None	2011	STR, AMP, AMC, FOX
SS-30	Peru	2012	AMP, AMC, SXT, CHL, SMX, TET
SS-31	Peru	2012	AMP, AMC, SXT, CHL, SMX, TET
SS-32	None	2012	STR, AMP, SXT, SMX, TET
SS-35	India	2012	STR, SXT, CIP, NAL, SMX, TET
SS-36	None	2012	STR, SXT, CIP, NAL, SMX, TET
SS-37	Haiti	2013	STR, AMP, SXT, SMX, TET
SS-38	Dominican Republic	2013	STR, AMP, SXT, SMX, TET
SS-39	None	2013	STR, SXT, CIP, NAL SMX, TET
SS-40	None	2013	STR, AMP, SXT, SMX, TET
SS-42	Dominican Republic	2014	STR, SXT, NAL, SMX, TET
SS-43	Mexico	2014	STR, SXT, NAL, SMX, TET

*Specimen identifications in the form of PSU-IDs were assigned to each *S. sonnei* isolate received from the Pennsylvania Department of Health.

†Travel history indicates where the patient is believed to have acquired the *S. sonnei* infection. ‘None’ indicates that the patient did not report travelling outside of the USA.

‡Antimicrobials listed indicate resistances previously reported [16]. STR, streptomycin; GEN, gentamicin; AMP, ampicillin; AMC, amoxicillin and clavulanic acid; FOX, cefoxitin; SXT, trimethoprim-sulfamethoxazole; SMX, sulfamethoxazole; TET, tetracycline; CHL, chloramphenicol; CIP, ciprofloxacin; NAL, nalidixic acid; AZM, azithromycin.

## Methods

### Bacterial strains and growth conditions

*S. sonnei* isolates sequenced in this study (Table 1) were submitted to the Pennsylvania Department of Health Bureauof Laboratories (BOL) in compliance with mandatory reporting regulations. AMR profiles in this earlier study [16] were determined using methods described by the Clinical and Laboratory Standards Institute [17], which is also available online (http://file.qums.ac.ir/repository/mmrc/CLSI2015.pdf).

Isolates chosen for the current study were resistant to at least three classes of antibiotics and included those associated with domestic (*n*=11) or overseas (*n*=11) acquired infections. After receiving bacterial isolates from the BOL, they were promptly inoculated from the shipping stabs onto sterile Lysogeny broth (LB) agar plates and grown overnight at 37 °C.

### DNA extraction, library preparation and WGS

For DNA extraction, a pure colony from each overnight agar culture was inoculated into 3 ml of LB for overnight growth at 37 °C in a shaking incubator at 300 r.p.m. DNA extraction was performed using a Wizard Genomic DNA kit (Promega), following the manufacturer’s instructions. DNA concentration was quantified in a Qubit 2.0 fluorometer using the Qubit dsDNA BR Assay kit (Thermo-Fisher) and DNA purity was checked using a A_260_/A_280_ purity ratio. Target DNA concentration was at or greater than 10 ng µl^−^^1^ and target purity ratio was greater than 1.8 and less than 2.2. The DNA of each strain was then diluted to a concentration of 0.2 ng µl^−^^1^. Sequencing libraries were prepared using the Nextera XT DNA Library Preparation Kit (Illumina) as per the manufacturer’s instructions. An Illumina MiSeq device was used to sequence the isolates, using 250 bp paired-end read length sequencing chemistry.

### Read assembly and quality control

Sequencing read quality was initially determined using FastQC 0.11.5 [18], followed by assembly using the SPAdes Genome Assembler Version 3.10.0 [19] with default parameters. Assembled genomes were then run in quast 4.5 [20] to determine the number of contigs, the N50 score and the total length of the assembled genome. Lastly, read coverage was calculated by aligning the reads of each strain to the reference genome, *S. sonnei* Ss046 (NCBI Accession Number NC_007384.1/CP_000038.1), using the Burrow-Wheeler Aligner 0.7.15 [21] and SAMtools 0.1.18 [22] to create a BAM file. Next, the SAMtools depth function was used to calculate average read coverage. Once collected, the generated parameters were compared to the values used for sequencing of *E. coli* by the Centers for Disease Control [23]. All quality control values are reported in Table S3. This process was also used to determine the quality of genomes downloaded from the NCBI website.

### AMR gene identification

Assembled genomes were screened using blast+ [24] against the Bacterial Antimicrobial Resistance Reference Gene Database (BARRGD) to identify AMR genes (BioProject number PRJNA313047, accessed May 2017). Genes with high nucleotide identity (>99 % identity) and high query coverage (>99 % coverage) were marked as present within the genome, and the genomes were next screened using the ResFinder 3.0 database [25], using default parameters (>90 % nucleotide identity and 60 % query coverage). Only genes identified by both BARRGD and ResFinder were used for comparisons of phenotypic results. Additionally, ResFinder (accessed 6 November 2017) was used to identify known chromosomal mutations that result in AMR phenotypes.

### Phylogenetic analysis

The Single Nucleotide Variant Phylogenomics (SNVPhyl) pipeline was used to perform SNP calling between the *S. sonnei* isolates and to construct phylogenetic trees [26]. All parameters were kept at default settings. *S. sonnei* Ss046 was used as the reference genome for all SNVPhyl runs. The output of SNVPhyl included a maximum-likelihood phylogenetic tree generated by PhyML, an SNP distance matrix table, and an SNP table with all called SNPs and their locations compared to the reference genome. Bootstrap values were calculated by PhyML 3.0 [27]. Select phylogenetic trees were visualized using Microreact [28].

### Identification of presumptive lineage alleles

Presumptive lineage alleles were identified by manually reviewing the SNVPhyl-generated SNP table. Only SNPs designated ‘Valid’ by the SNVPhyl workflow were considered. SNPs that occurred only in Lineage II or Lineage III strains were selected, and the genes containing these SNPs were extracted from the *S. sonnei* Ss046 genome. Through this process, 37 putative discriminatory alleles were identified. Next, *S. sonnei* genomes of known Global Lineage from the Holt collection (Table S2) were downloaded from the NCBI Sequence Read Archive (SRA) using the SRA Toolkit Version 2.8.1 [29]. The reads were assembled and read quality was checked using the methods described above. Additionally, SNVPhyl was used to perform SNP calling on these *S. sonnei* and to create a phylogenetic tree, segregating isolates by lineage. The assembled genomes were then aligned to the 37 presumptive lineage alleles using blast+. Allele sequences with 100 % nucleotide identity to all genomes of Lineage II or Lineage III *S. sonnei* were retained. This process narrowed the list of alleles from 37 to 17.

The ability of these alleles to classify isolates was further tested on 39 *S.sonnei* genomes (Table S2). These genomes were selected from public databases, with an emphasis on *S. sonnei* strains from different submitters, locations and time periods. The final collection included genomes from various continents including North and South America, Europe and Asia, and they were all sequenced between 2012 and 2017.

## Results

### Phylogenetic analysis of *S. sonnei* collection

**Fig. 1.**
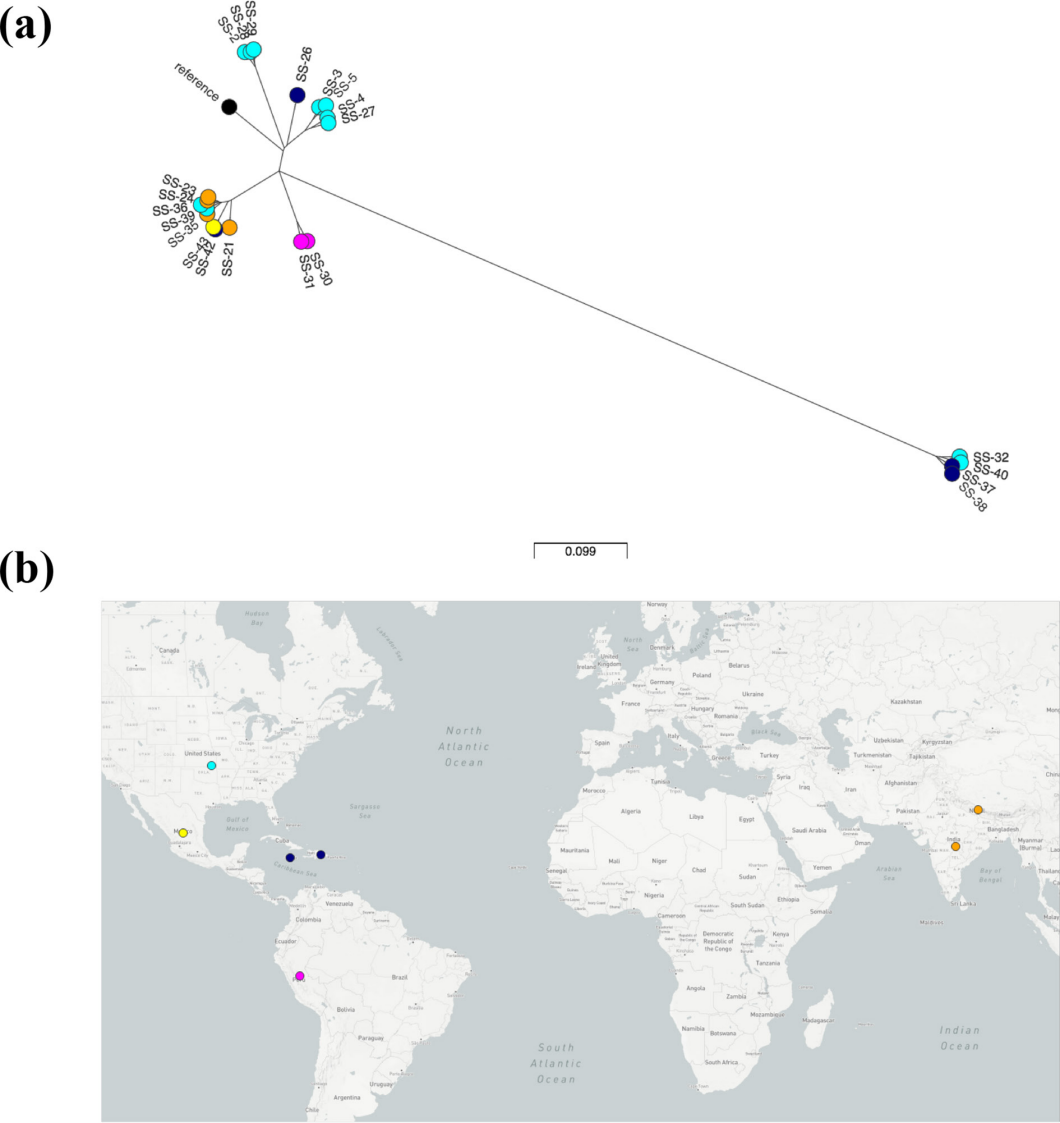
*S.sonnei* phylogenetic tree and presumed geographical source. This tree is a maximum likelihood tree generated using the GTR + gamma model and 1,000 bootstraps. The scale represents a branch length estimating the number of nucleotide substitutions per site. (a) *S. sonnei* phylogenetic tree generated by SNVPhyl using the reference genome *S. sonnei* Ss046. (b) Presumed geographical source of isolates obtained from the Pennsylvania Department of Health. The coloured circles of the tree correspond with the matching coloured circles on the world map. Colours designate region, but the locations of the points correspond to the country of origin rather than an exact geographical location. An interactive version of this output can be found at https://microreact.org/project/rJxfWLQdb.

**Fig. 2.**
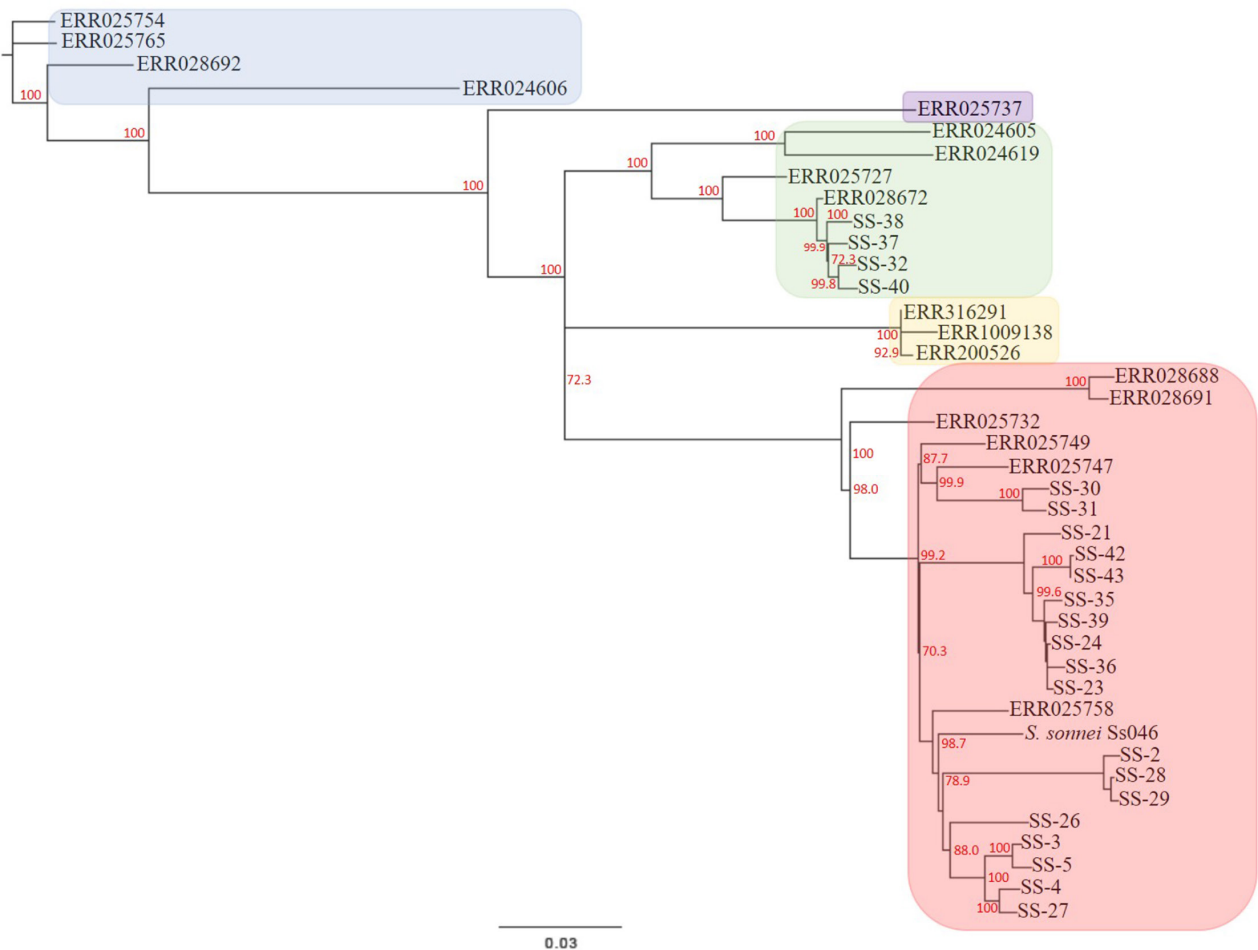
Global lineage analysis of the *S. sonnei* collection. A GTR gamma maximum-likelihood phylogenetic tree was generated by SNVPhyl to compare the Global Lineage strains to the *S. sonnei* collection, and bootstrap values were generated by reanalyzing the tree using PhyML. The scale depicts a branch length estimating the number of nucleotide substitutions per site.The blue highlighted region contains isolates in Lineage I, purple contains Lineage IV, green contains Lineage II, red contains Lineage III and yellow contains Lineage V.

SNP calling was performed after WGS of the 22 isolates obtained from the Pennsylvania Department of Health, and a maximum-likelihood tree was generated to visualize the relatedness of these *S. sonnei*. The *S. sonnei* isolates separated into two distinct clusters, separated by >850 SNPs, with isolates SS-32, SS-37, SS-38 and SS-40 segmenting distinctly from the remaining 18 isolates (Fig. 1). There was no evidence of clustering by geographical origin (https://microreact.org/project/rJxfWLQdb), as both clusters contained isolates that were acquired domestically and internationally. We next hypothesized that the clusters observed represented distinct lineages of *S. sonnei* that were previously described [8]. To test this, genomes of isolates from the Holt collection [8] representing Global Lineages I–IV, and three isolates of Lineage V [7] were downloaded. When analysed together, the Pennsylvania *S. sonnei* sequences segmented with isolates previously designated as Lineage II or III (Fig. 2), mirroring the two main clusters in Fig. 1(a). The four isolates from Pennsylvania mentioned above (SS-32, SS-37, SS-38 and SS-40) clustered with strains in Lineage II, and the other 18 isolates clustered with Lineage III.

### Global Lineage prediction gene allelic variants

**Fig. 3.**
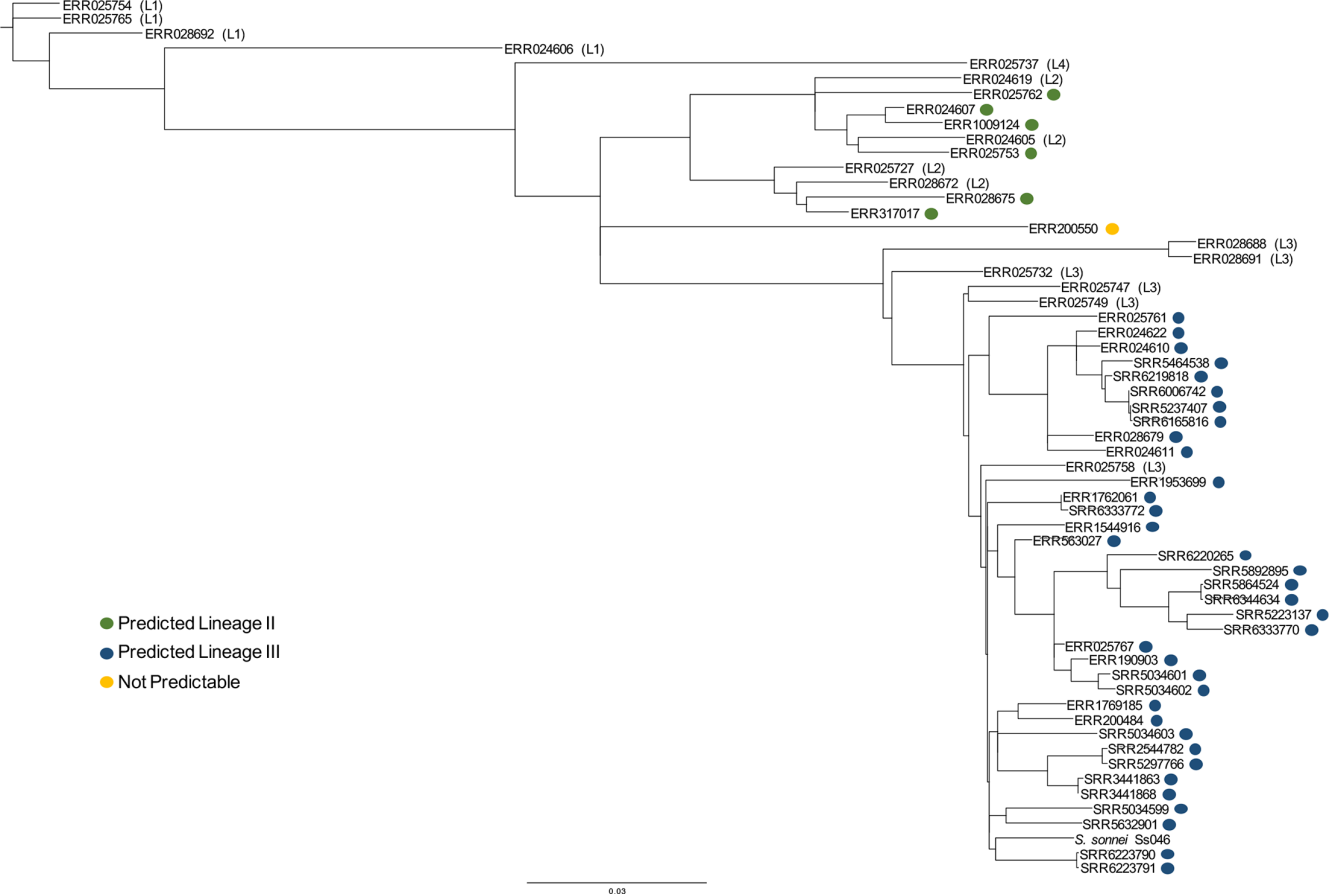
Determining the accuracy of *S. sonnei* lineage classification using presumptive lineage alleles. This tree is a maximum likelihood tree generated using the GTR + gamma model and 1,000 bootstraps. The scale represents a branch length estimating the number of nucleotide substitutions per site. Genomes downloaded from NCBI, including known global lineage isolates from the Holt collection, were aligned by SNVPhyl. The coloured dots represent the lineage designation predicted by the presumptive lineage alleles identified in this study.

Four putative Lineage II and 13 putative Lineage III allele sequences were initially identified (Table 2). To test their predictive accuracy, 39 selected *S. sonnei* genomes were downloaded from the NCBI SRA (Table S2). These genomes were checked for read quality and screened against the 17 presumptive lineage alleles. Three isolates carried all four Lineage II alleles and no Lineage III alleles, while 26 isolates carried only Lineage III alleles (Table 3). An additional seven isolates had 12 of 13 Lineage III alleles, and two isolates carried alleles distinct from those in our screen. Placing genomes in phylogenetic context (Fig. 3) revealed that our classification correctly identified the three Lineage II isolates and 26 of 33 Lineage III isolates; allowing for a one allele difference, all Lineage III isolates were identified.

**Table 2.** Presumptive Lineage alleles sequences

**Lineage II alleles**			
SNP location*	**Gene name**†	**Annotation**†	**Nucleotide change‡**
418633	ribD	bifunctional uracil reductase	418633T>G
2740056	der	ribosome biogenesis GTPase	2740056G>T
3515017	murA	UDP-*N*-acetylglucosamine 1-carboxyvinyltransferase	3515017G>A
4424328	purH	phosphoribosylaminoimidazole-carboxamideformyltransferase	4424328G>A
**Lineage III alleles**			
854671	ybiV	conserved hypothetical protein (Cof-type HAD-IIB family hydrolase)	854671C>T
3542336	yhcJ	putative enzyme (*N*-acetylmannosamine-6-phosphate 2-epimerase)	3542336T>G
3853204	dprA	putative DNA processing protein (DNA-processing protein DprA)	3853204G>A
4803842	deoA	thymidine phosphorylase	4803842G>A
592274	ybdH	putative oxidoreductase	592274T>C
3718965	malT	positive regulator of *mal* regulon	3718965A>G
3907787	yhiN	conserved hypothetical protein (aminoacetone oxidase family FAD-binding enzyme)	3907787A>G
4126099	pstA	high-affinity phosphate-specific transport protein	4126099T>C
4483455	dinF	DNA-damage-inducible protein F (MATE family efflux transporter)	4483455C>T
4621516	yjeF	conserved hypothetical protein (bifunctional ADP-dependent NAD(P)H-hydrate dehydratase/NAD(P)H-hydrate epimerase)	4621516T>C
4649181	sgaE	putative epimerase/aldolase (l-ribulose-5-phosphate 4-epimerase)	4649181T>C
4221555	recQ	ATP-dependent DNA helicase	4221555T>C
4189270	yifM	Uncharacterized conserved protein (TDP-*N*-acetylfucosamine lipid II *N*-acetylfucosaminyltransferase)	4189270T>G

*SNP location refers to the location of the presumptive lineage SNP in the reference genome *S. sonnei* Ss046 (NCBI Accession Number CP000038.1).

†Gene names were taken from the *S. sonnei* Ss046 annotation. Any ORFs designated as putative or hypothetical proteins were used as input for blast, and a putative function is included in parentheses based on the closest match to a gene annotated with a proposed function.

‡Nucleotide changes were identified during the SNP calling process and were further verified by the blast+ BTOP function. To read the SNP nomenclature, the first number indicates the location of the SNP in the reference genome (*S. sonnei* Ss046), the following letter is the nucleotide at that position and the letter after the ‘>’ is the nucleotide observed in the gene.

**Table 3.** Identification of presumptive lineage SNPs nucleotide percentage identity of the presumptive lineage identifying alleles represented by the colouring of the boxes Dark green represents 100 % nucleotide identity to presumptive Lineage II alleles and light green represents less than 100 %. Dark blue represents 100 % nucleotide identity to presumptive Lineage III alleles and light blue represents less than 100 %. The grey-shaded column on the right indicates the predicted Global Lineage for each strain. ‘NP’ indicates non-predictable strains and ‘NSS’ indicates isolates were were known or predicted to be ‘Not *Shigella sonnei*’. The EIEC isolate is an enteroinvasive *E. coli* sequenced by Pettengill *et al*. [30]

	ribD	der	murA	purH	ybiV	yhcJ	dprA	deoA	ybdH	malT	yhiN	pstA	dinF	yjeF	sgaE	recQ	yifM	**Lineage**
SRR5997370																		II
ERR317017																		II
ERR200550																		V
ERR1009124																		II
SRR6011659																		III
SRR5995965																		III
SRR5892895																		III
SRR5864524																		III
SRR5632901																		III
SRR5237407																		III
SRR5223137																		III
SRR5034601																		III
SRR5034599																		III
SRR3441863																		III
ERR563027																		III
ERR200484																		III
ERR190903																		III
SRR6344634																		III
SRR6333772																		III
SRR6333770																		III
SRR6223791																		III
SRR6223790																		III
SRR6220265																		III
SRR6219818																		III
SRR6165816																		III
SRR6006742																		III
SRR5464538																		III
SRR5297766																		III
SRR5034603																		III
SRR5034602																		III
SRR3441868																		III
SRR2544782																		III
ERR1953699																		III
ERR1769185																		III
ERR1762061																		III
ERR1544916																		III
ERR025750																		III
SRR5943575																		NSS
SRR5943576																		NSS
EIEC																		NSS
**S. boydii****Sb227**																		NSS
**S. boydii****ATCC 9210**																		NSS
**S. flexneri****2457**^**T**^																		NSS
**S. flexneri****Y394**																		NSS
***S. dysentariae*****1617**																		NSS
***S. dysentariae* Sd197**																		NSS

Three of the 39 isolates could not be classified using this method. Lineage V isolate ERR200550 carried one Lineage II and three Lineage III sequences and was separated from such strains in the phylogenetic tree (Fig. 3). Two other isolates, SRR5943575 and SRR5943576, carried neither Lineage II nor III alleles. SerotypeFinder [31] identified both as O7:H18, a serotype not associated with *S. sonnei*, suggesting that these isolates were misidentified as *Shigella*. It is noteworthy that none of the isolates from other *Shigella* species (*S. flexneri*, *S. dysentariae* and *S. boydii*) or the phenotypically similar enteroinvasive *E. coli* (SRR5943575 and SRR5943576) carried any of the presumptive lineage alleles.

### AMR in *S. sonnei* isolates

**Fig. 4.**
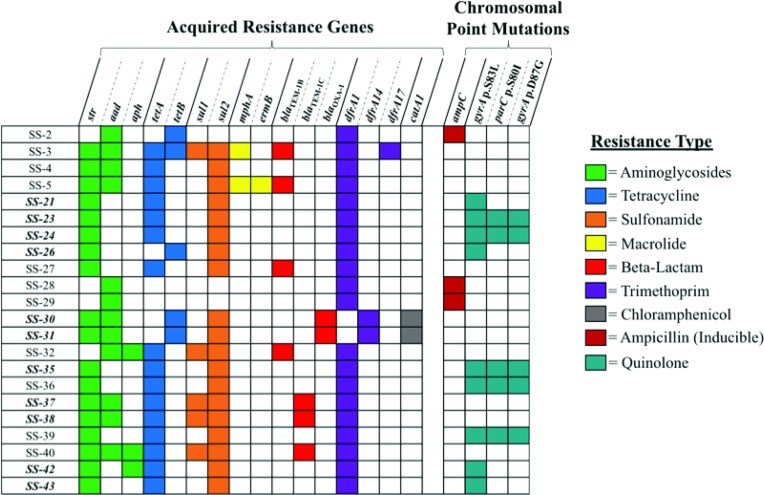
Antimicrobial resistance genes identified in *S. sonnei* sequences The strain designation is listed on the left, and resistance genes identified in one or more genomes are listed on the top. The bold and italic strain designations indicate *S. sonnei* isolates associated with international travel. Coloured squares indicate that the isolate carries a resistance gene with high identity and coverage (>99 %) to the query sequence. The colour of the square corresponds to the resistance type shown on the right side. All genes shown were identified by both BARRGD and ResFinder 3.0, with the exception being the chromosomal point mutations, which were only identified by ResFinder 3.0.

All 22 of the Pennsylvania isolates carried resistance genes for aminoglycosides and trimethoprim (Fig. 4, Table S4). Predicted resistances to chloramphenicol, macrolides, sulfonamides, beta-lactam antibiotics, tetracycline and quinolones were also identified within the collection. Notably, none of the Lineage II *S. sonnei* carried quinolone resistance determinants, and three of the four carried *bla*_TEM-1C_, while ampicillin resistance in Lineage III correlated with *bla*_TEM-1B_ or *bla*_OXA-1_. There was a 100 % correlation between genotype and phenotype for six of eight classes of antibiotics (Table 4), with discrepancies found for trimethoprim and aminoglycosides. For both of these, analyses identified resistance genes in all 22 isolates, but only 19 and 20 were phenotypically resistant to trimethoprim and aminoglycosides, respectively. The genes *strA*, *strB* and *aadA1* were identified in the two aminoglycoside-sensitive isolates (Table S4), SS-30 and SS-31, but *strA* was disrupted by *dfrA14* as previously described [32], and *aadA1* appeared intact with a single amino acid difference (Ala_4_→Val) compared to the reference sequence. The three trimethoprim-sensitive isolates, SS-2 SS-28 and SS-29, all carried apparently intact *dfrA1* genes.

**Table 4.** AMR genotype and phenotype comparisons

**Resistance type**	Phenotypic resistance*,†	**Genotypic resistance**	
		**Acquired**†	**Chromosomal mutations**†
Aminoglycosides	20 (91 %)	22 (100 %)	0 (0 %)
Beta-lactams	12 (55 %)	9 (41 %)	3 (14 %)
Sulfonamides	19 (86 %)	19 (86 %)	0 (0 %)
Trimethoprim	19 (86 %)	22 (100 %)	0 (0 %)
Chloramphenicol	2 (9 %)	2 (9 %)	0 (0 %)
Quinolones	9 (41 %)	0 (0 %)	9 (41 %)
Tetracycline	20 (91 %)	20 (91 %)	0 (0 %)
Macrolides‡	2 (10 %)	2 (10 %)	0 (0 %)

*The data were taken from a previous study [16]. The isolate was considered phenotypically resistant to a class of antibiotics if it was resistant to one or more antibiotic within the class.

†The number of *S. sonnei* within the collection of 22 isolates that were resistant to that particular antimicrobial class. The number in parentheses is the percentage of the total isolates that were resistant.

‡Only 20 out of 22 *S.sonnei* isolates were tested for *in vitro* macrolide resistance.

## Discussion

WGS analysis previously defined five lineages of *S. sonnei* from several geographical regions [7, 8, 33]. These studies, however, did not include *Shigella* from the USA, and to the best of our knowledge only two such analyses have been reported [9, 34]. The study of Chung The *et al*. [34] analysed the genomes of 14 isolates from the USA, and identified all as Lineage III. Additionally, 10 of these were predicted to be ciprofloxacin-resistant from their genome sequences. Similarly, *S. sonnei* from two outbreaks in California were all classified as Lineage III [9], and isolates from an outbreak localized to San Francisco were resistant to fluoroquinolones. These results align with the current study, as we report that 18 of the 22 *S.sonnei* isolates from Pennsylvania cluster into Lineage III, and nine of these isolates have point mutations associated with reduced susceptibility to fluoroquinolones [35]. The California study also identified within historical collections three Lineage II strains isolated between 1980 and 1987, and one Lineage I isolate from 1998. The work we report here indicates that Lineage II *S. sonnei* were circulating in the USA in 2013. Still, ours and the earlier studies collectively suggest human illness in the USA is primarily caused by Lineage III *S. sonnei*.

The adoption of WGS by public health laboratories for surveillance and outbreak investigation promotes the development of sequence-based tools for rapid characterization of bacterial pathogens. *S. sonnei* of various lineages are distinct from one another in traits relevant to public health such as antibiotic resistance and transmissibility [9], highlighting the importance of incorporating lineage identification into routine surveillance. With this in mind, the presumptive lineage allele sequences identified here could be explored as a way to rapidly classify isolates from the two most common worldwide lineages. Sangal *et al*. identified four gene sequences with lineage-specific SNPs and developed a lineage prediction tool using high-resolution melting analysis [36]. These alleles showed high specificity for identifying lineages, and one of the alleles they use, *deoA*, was also identified by the methods used in our study. Our approach, however, suggests that this method needs to be revisited in light of reports of Lineage V [7], as at least the ERR200250 isolate analysed here carries the same *deoA* allele as Lineage II isolates.

WGS is increasingly used to predict AMR, with the ultimate goal of replacing phenotypic testing. Limited studies have investigated the correlation between genomic predictions and phenotype for *S. sonnei*. In one study, there was perfect concordance between these methods for beta-lactam, sulfonamide, chloramphenicol, quinolone, tetracycline and macrolide resistance, and a strong but lower (86–91 %) concordance for aminoglycoside and trimethoprim resistance [9]. A strong correlation between these two methods has also been reported for other foodborne pathogens including *Salmonella* and *E. coli* [11–14]. The results of Kozyreva *et al*. [9] and Sadouki *et al*. [15] represent the only other reports we are aware of that test the predictive ability of WGS using *S. sonnei* isolates. Interestingly, that former study reported that 100 % of isolates carrying an AMR gene were phenotypically resistant to the corresponding antibiotic, while 7 % of isolates were phenotypically resistant, although they lacked a gene known to confer this trait. By contrast, all discrepancies in the Soudouki *et al*. [15] study were for genotypically resistant but phenotypically sensitive isolates, which our work found as well. Some of our isolates carried apparently intact genes, suggesting the observed phenotype may be due to the lack of gene expression. Another possibility, as noted previously for *Salmonella* [14], is that breakpoint values for interpreting antimicrobial susceptibility testing may not align with the resistance conferred by all AMR genes. Of note, the Clinical and Laboratory Standards Institute has no *Shigella* species minimum inhibitory concentration breakpoints for aminoglycosides and trimethoprim as they are not utilized clinically [17]. Whether the lack of correspondence between genetic predictions and the *in vitro* testing results from a lack of standardized methods deserves further investigation. Future studies are also needed to resolve whether these findings are also applicable to drug-susceptible *S. sonnei*.

AMR in *S. sonnei* is important to examine due to its human health implications, and also because of its proposed influence on the evolution of this strain. For example, acquisition of certain acquired AMR genes and chromosomal mutations conferring resistance was suggested to occur after lineage divergence [8, 33], and AMR may be responsible in part for the increased prevalence of *S. sonnei* infections in developed regions over *S. flexneri* [6]. As mentioned previously, within our collection only Lineage III isolates carried resistance to quinolones and macrolides, the two drugs typically used to treat *Shigella* infections in the USA. This observation aligns with previous literature [7–9, 33, 34]. Quinolone resistance in Lineage III *S. sonnei* has also been reported to be primarily due to chromosomal mutations, which we observed in the current study. It has been hypothesized that Lineage III *S. sonnei* are more likely to acquire both chromosomal mutations and genes that result in AMR due to selective pressures, regardless of geographical origin [33].

In summary, our study increases our understanding of *S. sonnei* associated with illnesses reported to public health authorities in the USA and highlights the role of WGS in elucidating phenotypic/genotypic AMR correlation. Incorporating molecular tools for lineage prediction into active surveillance may prove useful for monitoring the spread of AMR in *S. sonnei* and for identifying the possible rise of new variants.

## Data Bibliography

1. Food and Drug Administration Center for Food Safety and Applied Nutrition. NCBI BioProject PRJNA273284. (2017).
  2. Yang F., Yang J., Zhang X., Chen L., Jiang Y., Yan Y., Tang X., Wang J., Xiong Z., Dong J., Xue Y., Zhu Y., Xu X., Sun L., Chen S., Nie H., Peng J., Xu J., Wang Y., Yuan Z., Wen Y., Yao Z., Shen Y., Qiang B., Hou Y., Yu J. and Jin Q. NCBI Genomes: CP_000038.1. (2004).

## Supplementary Data

Supplementary File 1
