# Supplementary material for: Use of whole genome sequencing in surveillance for antimicrobial-resistant Shigella sonnei infections acquired from domestic and international sources
Source: Microb Genom. 2019 May 17;5(5):e000270. doi: 10.1099/mgen.0.000270 (PMC6562246; doi:10.1099/mgen.0.000270)
Supplement: Supplementary File 1 [file mgen-5-270-s001.pdf]

Supplementary Materials

Supplementary Table 1: Isolates Sequenced in this Study

PSU ID[a]	BioSample	SRR Accession Number
SS-2	SAMN07571653	SRR6114360
SS-3	SAMN07571594	SRR5990599
SS-4	SAMN07571572	SRR6114439
SS-5	SAMN07571571	SRR6114438
SS-21	SAMN07571584	SRR6219657
SS-23	SAMN07571602	SRR6219818
SS-24	SAMN07571634	SRR6220146
SS-26	SAMN07571636	SRR6220069
SS-27	SAMN07571633	SRR6220295
SS-28	SAMN07571614	SRR6220265
SS-29	SAMN07571612	SRR6220297
SS-30	SAMN07571618	SRR6219723
SS-31	SAMN07571616	SRR6219731
SS-32	SAMN07571648	SRR6219745
SS-35	SAMN07571651	SRR6219726
SS-36	SAMN07571565	SRR5990584
SS-37	SAMN07571566	SRR6114364
SS-38	SAMN07571562	SRR5990593
SS-39	SAMN07571567	SRR6113231
SS-40	SAMN07571563	SRR5990590
SS-42	SAMN07571568	SRR6113305
SS-43	SAMN07571573	SRR6113189

[a]All sequences were uploaded to NCBI under BioProject Number PRJNA273284.

Supplementary Table 2: Additional Sequences Used in this Study.

Isolate and Accession Number	Figure 2	Figure 3	Table 3	Bioproject	Reference
**Reference sequence**					
S. sonnei Ss046 (CP000038.1)				PRJNA13151	(16)
**Other *Shigella sonnei* isolates**					
ERR025754	X	X		PRJEB2128	(17)
ERR025765	X	X		PRJEB2128	(17)
ERR028692	X	X		PRJEB2128	(17)
ERR024606	X	X		PRJEB2128	(17)
ERR025737	X	X		PRJEB2128	(17)
ERR024619		X		PRJEB2128	(17)
ERR025762		X		PRJEB2128	(17)
ERR024607		X		PRJEB2128	(17)
ERR1009124		X	X	PRJEB9146	
ERR024605	X	X		PRJEB2128	(17)

ERR024619	X			PRJEB2128	(17)
ERR025753		X		PRJEB2128	(17)
ERR025727	X	X		PRJEB2128	(17)
ERR028672	X	X		PRJEB2128	(17)
ERR028675		X		PRJEB2128	(17)
ERR317017		X	X	PRJEB3255	
ERR200550		X	X	PRJEB2846	(7)
ERR028688	X	X		PRJEB2128	(17)
ERR028691	X	X		PRJEB2128	(17)
ERR025732	X			PRJEB2128	(17)
ERR025747	X			PRJEB2128	(17)
ERR025749	X	X		PRJEB2128	(17)
ERR025761		X		PRJEB2128	(17)
ERR024622		X		PRJEB2128	(17)
ERR024610		X		PRJEB2128	(17)
SRR5464538		X	X	PRJNA218110	
SRR6219818		X	X	PRJNA273284	
SRR6006742		X	X	PRJNA278886	
SRR5237407		X		PRJNA278886	
SRR6165816		X	X	PRJNA278886	
ERR028679		X		PRJEB2128	(17)
ERR024611		X		PRJEB2128	(17)
ERR025758	X	X		PRJEB2128	(17)
ERR1953699		X	X	PRJEB20541	
ERR1762061		X	X	PRJEB18660	
SRR6333772		X		PRJNA218110	
ERR1544916		X	X	PRJEB14038	
ERR563027		X		PRJEB2975	
SRR6220265		X		PRJNA273284	
SRR5892895		X		PRJNA218110	
SRR5864524		X		PRJNA218110	
SRR6344634		X		PRJNA218110	
SRR5223137		X		PRJNA218110	
SRR6333770		X		PRJNA218110	
ERR025767		X		PRJEB2128	(17)
ERR190903		X		PRJEB2975	
SRR5034601		X		PRJNA315192	
SRR5034602		X		PRJNA315192	
ERR1769185		X		PRJEB18610	
ERR200484		X		PRJEB2846	
SRR2544782		X		PRJNA278886	
SRR5297766		X		PRJNA218110	
SRR3441863		X		PRJNA300887	
SRR3441868		X		PRJNA300887	
SRR5034599		X		PRJNA315192	
SRR5632901		X		PRJNA218110	

SRR6223790		X		PRJNA415957	
SRR6223791		X		PRJNA415957	
ERR316291	X			PRJEB2846	(7)
ERR1009138	X			PRJEB9146	(7)
ERR200526	X			PRJEB2846	(7)
SRR5997370			X	PRJNA218110	
SRR6011659			X	PRJNA218110	
SRR5995965			X	PRJNA218110	
SRR5892895			X	PRJNA218110	
SRR5864524			X	PRJNA218110	
SRR5632901			X	PRJNA218110	
SRR5237407			X	PRJNA278886	
SRR5223137			X	PRJNA218110	
SRR5034601			X	PRJNA315192	
SRR5034599			X	PRJNA315192	
SRR3441863			X	PRJNA300887	(18)
ERR563027			X	PRJEB2975	
ERR200484			X	PRJEB2846	
ERR190903			X	PRJEB2975	
SRR6344634			X	PRJNA218110	
SRR6333772			X	PRJNA218110	
SRR6333770			X	PRJNA218110	
SRR6223791			X	PRJNA415957	
SRR6223790			X	PRJNA415957	
SRR6220265			X	PRJNA273284	
SRR5297766			X	PRJNA218110	
SRR5034603			X	PRJNA315192	
SRR5034602			X	PRJNA315192	
SRR3441868			X	PRJNA300887	(18)
SRR2544782			X	PRJNA278886	
ERR1769185			X	PRJEB18610	
ERR025750			X	PRJEB2128	(17)
SRR5943575			X	PRJNA389557	
SRR5943576			X	PRJNA389557	
E. coli strain CFSAN029787 (CP011416.1)			X	PRJNA243331	(19)
S. boydii Sb227 (NC_007613.1)			X	PRJNA224116	(16)
S. boydii ATCC 9210 (CP011511.1)			X	PRJNA284097	
S. flexneri 2457T (AE014073.1)			X	PRJNA408	(20)
S. flexneri Y394 (CP020753.1)			X	PRJNA382451	(21)
S. dysentariae 1617 (CP006736.1)			X	PRJNA218020	(22)
S. dysentariae Sd197 (NC_007606.1)			X	PRJNA58213	(16)

[a]Isolate reference listed when available.

Supplementary Table 3: Quality Control Values of Sequenced *S. sonnei*

PSU-ID	Coverage[a]	Reads[b]	# of Contigs[c]	N50[c]	Total Length[c]
E. coli[d]	>40	-	<400	>100,000	4500000-6000000
SS-2	48.1	1,046,470	574	25093	4729633
SS-3	32.6	1,040,270	636	24748	4824973
SS-4	47.8	983,998	559	25880	4563795
SS-5	39.6	881,762	596	26294	4604296
SS-21	96.5	2,219,142	537	25394	4487283
SS-23	75.9	1,614,176	539	25390	4491508
SS-24	111.6	2,372,628	581	24529	4661099
SS-26	99.5	2,167,098	572	25933	4714253
SS-27	66.7	1,410,486	528	25985	4605355
SS-28	107.6	2,315,582	561	27121	4690652
SS-29	67.7	1,548,124	552	27163	4690092
SS-30	56.6	1,262,222	618	25979	4778593
SS-31	103.45	2,203,584	547	25949	4615949
SS-32	48.2	1,023,846	515	28556	4680028
SS-35	41.6	854,052	475	25452	4489651
SS-36	62.8	1,329,678	558	25398	4563502
SS-37	42.9	904,156	562	27344	4752313
SS-38	58.6	1,267,340	551	28601	4688007
SS-39	76.9	1,617,026	600	25398	4485717
SS-40	69.2	1,511,954	560	25901	4751864
SS-42	54.9	1,111,912	1074	23416	4653788
SS-43	54.6	1,107,538	558	25907	4668418

[a]Coverage, a calculation of how the sequencing reads map to a reference genome, was generated by aligning the reads to a reference genome (*S. sonnei* Ss046) to create a BAM file. This BAM file was then input into the Samtools depth function to calculate coverage.
[b]The reads were reported by the Illumina MiSeq instrument after sequencing was completed.
[c]QUAST 4.5 was used to calculate the number of contigs, the N50 score, and the total length of the assembled genome. These parameters are indicative of the quality of the assembly performed by the SPAdes Genome Assembler Version 3.10.0.
[d]The values listed are parameters used by the CDC to determine sequence quality of *E. coli* genomes.

Supplementary Table 4: Results of Resfinder and BARRGD searches

	Resistance gene	Identity	Query/HSP	Contig	Position in contig	Phenotype	Accession no.	Mutation	Nucleotide change	Amino acid change	Resistance	PMID
SS-2	aadA1	100	789/789	SS-2_NODE_38_length_33744_cov_25.7775	29397..30185	Aminoglycoside resistance	JQ480156	ampC promoter n.-42C>T	C → T	Promoter mutations	B-lactam resistance	21653764
	tet(B)	100	1206/1206	SS-2_NODE_284_length_2620_cov_10.9824	245..1450	Tetracycline resistance	AF326777					
	dfrA1	100	474/474	SS-2_NODE_38_length_33744_cov_25.7775	30862..31335	Trimethoprim resistance	X00926					
SS-3	aac(3)-IId	99.88	861/861	SS-3_NODE_335_length_1469_cov_16.4888	244..1104	Aminoglycoside resistance	EU022314					
	aadA1	100	789/789	SS-3_NODE_208_length_5554_cov_25.3167	3396..4184	Aminoglycoside resistance	JQ480156					
	strB	100	837/837	SS-3_NODE_7_length_65365_cov_23.508	63571..64407	Aminoglycoside resistance	M96392					
	strA	100	804/804	SS-3_NODE_7_length_65365_cov_23.508	64407..65210	Aminoglycoside resistance	AF321551					
	aadA5	100	789/789	SS-3_NODE_142_length_10219_cov_11.311	7799..8587	Aminoglycoside resistance	AF137361					
	blaTEM-1B	100	861/861	SS-3_NODE_7_length_65365_cov_23.508	57713..58573	Beta-lactam resistance	JF910132					
	mph(A)	100	906/906	SS-3_NODE_142_length_10219_cov_11.311	197..1102	Macrolide resistance	D16251					
	sul2	100	816/816	SS-3_NODE_371_length_944_cov_187.554	33..848	Sulphonamide resistance	GQ421466					
	sul1	100	927/927	SS-3_NODE_142_length_10219_cov_11.311	6413..7339	Sulphonamide resistance	CP002151					
	tet(B)	100	1206/1206	SS-3_NODE_291_length_2633_cov_10.7873	58..1263	Tetracycline resistance	AF326777					
	tet(A)	100	1200/1200	SS-3_NODE_7_length_65365_cov_23.508	61036..62235	Tetracycline resistance	AJ517790					
	dfrA1	100	474/474	SS-3_NODE_208_length_5554_cov_25.3167	2246..2719	Trimethoprim resistance	X00926					
	dfrA17	100	474/474	SS-3_NODE_142_length_10219_cov_11.311	8718..9191	Trimethoprim resistance	FJ460238					
SS-4	aadA1	100	789/789	SS-4_NODE_55_length_25371_cov_24.6683	21045..21833	Aminoglycoside resistance	JQ480156					
	strB	100	837/837	SS-4_NODE_162_length_8528_cov_7.85192	56..892	Aminoglycoside resistance	M96392					
	strA	100	804/804	SS-4_NODE_162_length_8528_cov_7.85192	892..1695	Aminoglycoside resistance	AF321551					
	sul2	100	816/816	SS-4_NODE_162_length_8528_cov_7.85192	1756..2571	Sulphonamide resistance	GQ421466					
	tet(A)	100	1200/1172	SS-4_NODE_162_length_8528_cov_7.85192	5950..7121	Tetracycline resistance	AJ517790					
	dfrA1	100	474/474	SS-4_NODE_55_length_25371_cov_24.6683	22510..22983	Trimethoprim resistance	X00926					
SS-5	strA	100	804/684	SS-5_NODE_162_length_8517_cov_99.2448	1..684	Aminoglycoside resistance	AF321551					
	aadA1	100	789/789	SS-5_NODE_37_length_33702_cov_20.1774	29376..30164	Aminoglycoside resistance	JQ480156					
	strB	100	837/837	SS-5_NODE_162_length_8517_cov_99.2448	684..1520	Aminoglycoside resistance	M96392					
	blaTEM-1B	100	861/861	SS-5_NODE_138_length_10664_cov_27.2856	744..1604	Beta-lactam resistance	JF910132					
	mph(A)	100	906/906	SS-5_NODE_241_length_3454_cov_31.453	2354..3259	Macrolide resistance	D16251					
	erm(B)	99.86	738/738	SS-5_NODE_273_length_1987_cov_7.85806	420..1157	Macrolide resistance	JN899585					
	sul2	100	816/816	SS-5_NODE_162_length_8517_cov_99.2448	7395..8210	Sulphonamide resistance	GQ421466					
	tet(A)	100	1200/1172	SS-5_NODE_162_length_8517_cov_99.2448	2856..4027	Tetracycline resistance	AJ517790					
	dfrA1	100	474/474	SS-5_NODE_37_length_33702_cov_20.1774	30841..31314	Trimethoprim resistance	X00926					
SS-21	strA	99.88	804/804	SS21_NODE_162_length_8517_cov_2034.54	2556..3359	Aminoglycoside resistance	AF321551	gyrA p.S83L	TCG → TTG	S → L	Quinolones,Fluoroqui	15848289
	strB	100	837/837	SS21_NODE_162_length_8517_cov_2034.54	3359..4195	Aminoglycoside resistance	M96392					
	sul2	100	816/816	SS21_NODE_162_length_8517_cov_2034.54	1680..2495	Sulphonamide resistance	GQ421466					
	tet(A)	100	1200/1172	SS21_NODE_162_length_8517_cov_2034.54	5520..6691	Tetracycline resistance	AJ517790					
	dfrA1	100	474/474	SS21_NODE_38_length_32995_cov_58.5561	30062..30535	Trimethoprim resistance	X00926					
SS-23	strA	99.88	804/804	SS23_NODE_161_length_8528_cov_336.337	1350..2153	Aminoglycoside resistance	AF321551	parC p.S80I	AGC → ATC	S → I	Quinolones,Fluoroqui	15848289
	strB	100	837/837	SS23_NODE_161_length_8528_cov_336.337	2153..2989	Aminoglycoside resistance	M96392	gyrA p.S83L	TCG → TTG	S → L	Quinolones,Fluoroqui	15848289
	sul2	100	816/816	SS23_NODE_161_length_8528_cov_336.337	474..1289	Sulphonamide resistance	GQ421466	gyrA p.D87G	GAC → GGC	D → G	Quinolones,Fluoroqui	15848289
	tet(A)	100	1200/1172	SS23_NODE_161_length_8528_cov_336.337	4325..5496	Tetracycline resistance	AJ517790					
	dfrA1	99.79	474/474	SS23_NODE_38_length_32995_cov_47.1269	30062..30535	Trimethoprim resistance	X00926					
SS-24	strA	99.88	804/804	SS24_NODE_163_length_8528_cov_352.472	2428..3231	Aminoglycoside resistance	AF321551	parC p.S80I	AGC → ATC	S → I	Quinolones,Fluoroqui	15848289
	strB	100	837/837	SS24_NODE_163_length_8528_cov_352.472	3231..4067	Aminoglycoside resistance	M96392	gyrA p.S83L	TCG → TTG	S → L	Quinolones,Fluoroqui	15848289
	sul2	100	816/816	SS24_NODE_163_length_8528_cov_352.472	1552..2367	Sulphonamide resistance	GQ421466	gyrA p.D87G	GAC → GGC	D → G	Quinolones,Fluoroqui	15848289
	tet(A)	100	1200/1172	SS24_NODE_163_length_8528_cov_352.472	5403..6574	Tetracycline resistance	AJ517790					
	dfrA1	99.79	474/474	SS24_NODE_39_length_32995_cov_64.8099	30062..30535	Trimethoprim resistance	X00926					
SS-26	aadA1	100	789/789	SS26_NODE_195_length_5556_cov_80.447	3398..4186	Aminoglycoside resistance	JQ480156	gyrA p.S83L	TCG → TTG	S → L	Quinolones,Fluoroqui	15848289
	strB	97.16	837/670	SS26_NODE_300_length_1394_cov_1.59353	725..1394	Aminoglycoside resistance	M96392					
	sul2	100	816/816	SS26_NODE_5_length_72031_cov_28.4438	2048..2863	Sulphonamide resistance	GQ421466					
	tet(B)	100	1206/1206	SS26_NODE_235_length_3818_cov_27.2479	1326..2531	Tetracycline resistance	AF326777					
	dfrA1	100	474/474	SS26_NODE_195_length_5556_cov_80.447	2248..2721	Trimethoprim resistance	X00926					
SS-27	aadA1	100	789/789	SS27_NODE_36_length_34096_cov_45.3026	29573..30361	Aminoglycoside resistance	JQ480156					
	strB	100	837/837	SS27_NODE_160_length_8572_cov_41.5387	3620..4456	Aminoglycoside resistance	M96392					
	strA	100	804/804	SS27_NODE_160_length_8572_cov_41.5387	4456..5259	Aminoglycoside resistance	AF321551					
	blaTEM-1B	100	861/861	SS27_NODE_2_length_89309_cov_16.6015	21081..21941	Beta-lactam resistance	JF910132					
	sul2	100	816/816	SS27_NODE_160_length_8572_cov_41.5387	5320..6135	Sulphonamide resistance	GQ421466					
	tet(A)	100	1200/1172	SS27_NODE_160_length_8572_cov_41.5387	1113..2284	Tetracycline resistance	AJ517790					

	dfrA1	100	474/474	SS27_NODE_36_length_34096_cov_45.3026	31038..31511	Trimethoprim resistance	X00926					
SS-28	aadA1	100	789/789	SS28_NODE_39_length_33744_cov_56.302	29397..30185	Aminoglycoside resistance	JQ480156	ampC promoter n.-42C>T	C → T	Promoter mutations	B-lactam resistance	21653764
	dfrA1	100	474/474	SS28_NODE_39_length_33744_cov_56.302	30862..31335	Trimethoprim resistance	X00926					
SS-29	aadA1	100	789/789	SS29_NODE_38_length_33744_cov_36.5734	29397..30185	Aminoglycoside resistance	JQ480156	ampC promoter n.-42C>T	C → T	Promoter mutations	B-lactam resistance	21653764
	dfrA1	100	474/474	SS29_NODE_38_length_33744_cov_36.5734	30862..31335	Trimethoprim resistance	X00926					
SS-30	strA	100	804/529	SS30_NODE_188_length_6895_cov_141.423	1350..1878	Aminoglycoside resistance	AF321551					
	strB	100	837/837	SS30_NODE_188_length_6895_cov_141.423	2721..3557	Aminoglycoside resistance	M96392					
	aadA1	99.75	792/792	SS30_NODE_154_length_9027_cov_23.8816	8029..8820	Aminoglycoside resistance	JQ414041					
	blaOXA-1	100	831/831	SS30_NODE_154_length_9027_cov_23.8816	7086..7916	Beta-lactam resistance	J02967					
	catA1	99.85	660/660	SS30_NODE_154_length_9027_cov_23.8816	351..1010	Phenicol resistance	V00622					
	sul2	100	816/816	SS30_NODE_188_length_6895_cov_141.423	474..1289	Sulphonamide resistance	GQ421466					
	tet(B)	100	1206/1206	SS30_NODE_143_length_9887_cov_53.0163	2180..3385	Tetracycline resistance	AF326777					
	dfrA14	99.79	483/483	SS30_NODE_188_length_6895_cov_141.423	1889..2371	Trimethoprim resistance	DQ388123					
SS-31	aadA1	99.75	792/792	SS31_NODE_78_length_19916_cov_227.144	10557..11348	Aminoglycoside resistance	JQ414041					
	strA	100	804/529	SS31_NODE_181_length_6895_cov_111.903	1275..1803	Aminoglycoside resistance	AF321551					
	strB	100	837/837	SS31_NODE_181_length_6895_cov_111.903	2646..3482	Aminoglycoside resistance	M96392					
	blaOXA-1	100	831/831	SS31_NODE_78_length_19916_cov_227.144	11461..12291	Beta-lactam resistance	J02967					
	catA1	99.85	660/660	SS31_NODE_78_length_19916_cov_227.144	18367..19026	Phenicol resistance	V00622					
	sul2	100	816/816	SS31_NODE_181_length_6895_cov_111.903	399..1214	Sulphonamide resistance	GQ421466					
	tet(B)	100	1206/1206	SS31_NODE_78_length_19916_cov_227.144	6503..7708	Tetracycline resistance	AF326777					
	dfrA14	99.79	483/483	SS31_NODE_181_length_6895_cov_111.903	1814..2296	Trimethoprim resistance	DQ388123					
SS-32	aadA1	100	792/792	SS32_NODE_124_length_13225_cov_5.90029	3274..4065	Aminoglycoside resistance	JX185132					
	aph(3'')-Ib	100	804/804	SS32_NODE_5_length_74081_cov_6.74372	62592..63395	Aminoglycoside resistance	AF321551					
	aph(6)-Id	100	837/837	SS32_NODE_5_length_74081_cov_6.74372	63395..64231	Aminoglycoside resistance	M28829					
	blaTEM-1C	100	861/861	SS32_NODE_156_length_9012_cov_6.34811	7806..8666	Beta-lactam resistance	FJ560503					
	sul1	100	927/927	SS32_NODE_124_length_13225_cov_5.90029	4483..5409	Sulphonamide resistance	CP002151					
	sul2	100	816/816	SS32_NODE_5_length_74081_cov_6.74372	61716..62531	Sulphonamide resistance	GQ421466					
	tet(A)	100	1200/1200	SS32_NODE_156_length_9012_cov_6.34811	842..2041	Tetracycline resistance	AJ517790					
	dfrA1	100	474/474	SS32_NODE_124_length_13225_cov_5.90029	2708..3181	Trimethoprim resistance	JQ690541					
SS-35	strA	99.88	804/804	SS35_NODE_159_length_8528_cov_33.654	2428..3231	Aminoglycoside resistance	AF321551	parC p.S80I	AGC → ATC	S → I	Quinolones,Fluoroqui	15848289
	strB	100	837/837	SS35_NODE_159_length_8528_cov_33.654	3231..4067	Aminoglycoside resistance	M96392	gyrA p.S83L	TCG → TTG	S → L	Quinolones,Fluoroqui	15848289
	sul2	100	816/816	SS35_NODE_159_length_8528_cov_33.654	1552..2367	Sulphonamide resistance	GQ421466	gyrA p.D87G	GAC → GGC	D → G	Quinolones,Fluoroqui	15848289
	tet(A)	100	1200/1172	SS35_NODE_159_length_8528_cov_33.654	5403..6574	Tetracycline resistance	AJ517790					
	dfrA1	99.79	474/474	SS35_NODE_38_length_32995_cov_23.9069	30062..30535	Trimethoprim resistance	X00926					
SS-36	strA	99.88	804/804	SS-36_NODE_160_length_8528_cov_47.9821	2428..3231	Aminoglycoside resistance	AF321551	parC p.S80I	AGC → ATC	S → I	Quinolones,Fluoroqui	15848289
	strB	100	837/837	SS-36_NODE_160_length_8528_cov_47.9821	3231..4067	Aminoglycoside resistance	M96392	gyrA p.S83L	TCG → TTG	S → L	Quinolones,Fluoroqui	15848289
	sul2	100	816/816	SS-36_NODE_160_length_8528_cov_47.9821	1552..2367	Sulphonamide resistance	GQ421466	gyrA p.D87G	GAC → GGC	D → G	Quinolones,Fluoroqui	15848289
	tet(A)	99.91	1200/1172	SS-36_NODE_160_length_8528_cov_47.9821	5403..6574	Tetracycline resistance	AJ517790					
	dfrA1	99.79	474/474	SS-36_NODE_39_length_32849_cov_16.7053	29989..30462	Trimethoprim resistance	X00926					
SS-37	strB	100	837/837	SS-37_NODE_132_length_12031_cov_4.23522	1782..2618	Aminoglycoside resistance	M96392					
	aadA1	100	792/792	SS-37_NODE_174_length_7192_cov_3.48577	3273..4064	Aminoglycoside resistance	JX185132					
	strA	100	804/804	SS-37_NODE_132_length_12031_cov_4.23522	979..1782	Aminoglycoside resistance	AF321551					
	blaTEM-1C	100	861/861	SS-37_NODE_135_length_11788_cov_3.41266	347..1207	Beta-lactam resistance	FJ560503					
	sul2	100	816/816	SS-37_NODE_132_length_12031_cov_4.23522	103..918	Sulphonamide resistance	GQ421466					
	sul1	100	927/927	SS-37_NODE_174_length_7192_cov_3.48577	4482..5408	Sulphonamide resistance	CP002151					
	tet(A)	100	1200/1200	SS-37_NODE_135_length_11788_cov_3.41266	6972..8171	Tetracycline resistance	AJ517790					
	dfrA1	100	474/474	SS-37_NODE_174_length_7192_cov_3.48577	2707..3180	Trimethoprim resistance	JQ690541					
SS-38	aadA1	100	792/792	NODE_120_length_13330_cov_37.404	3377..4168	Aminoglycoside resistance	JX185132					
	strA	100	804/804	NODE_2_length_82535_cov_10.1199	71075..71878	Aminoglycoside resistance	AF321551					
	strB	100	837/837	NODE_2_length_82535_cov_10.1199	71878..72714	Aminoglycoside resistance	M96392					
	blaTEM-1C	100	861/861	NODE_2_length_82535_cov_10.1199	347..1207	Beta-lactam resistance	FJ560503					
	sul1	100	927/927	NODE_120_length_13330_cov_37.404	4586..5512	Sulphonamide resistance	CP002151					
	sul2	100	816/816	NODE_2_length_82535_cov_10.1199	70199..71014	Sulphonamide resistance	GQ421466					
	tet(A)	100	1200/1200	NODE_2_length_82535_cov_10.1199	6972..8171	Tetracycline resistance	AJ517790					
	dfrA1	100	474/474	NODE_120_length_13330_cov_37.404	2811..3284	Trimethoprim resistance	JQ690541					
SS-39	strB	100	837/837	SS-39_NODE_162_length_8528_cov_45.1112	3620..4456	Aminoglycoside resistance	M96392	parC p.S80I	AGC → ATC	S → I	Quinolones,Fluoroqui	15848289
	strA	99.88	804/804	SS-39_NODE_162_length_8528_cov_45.1112	4456..5259	Aminoglycoside resistance	AF321551	gyrA p.S83L	TCG → TTG	S → L	Quinolones,Fluoroqui	15848289
	sul2	100	816/816	SS-39_NODE_162_length_8528_cov_45.1112	5320..6135	Sulphonamide resistance	GQ421466	gyrA p.D87G	GAC → GGC	D → G	Quinolones,Fluoroqui	15848289

	tet(A)	100	1200/1172	SS-39_NODE_162_length_8528_cov_45.1112	1113..2284	Tetracycline resistance	AJ517790					
	dfrA1	99.79	474/474	SS-39_NODE_39_length_32995_cov_40.5527	30062..30535	Trimethoprim resistance	X00926					
SS-40	aadA1	100	792/792	NODE_128_length_13170_cov_11.1336	3274..4065	Aminoglycoside resistance	JX185132					
	aph(3')-Ia	99.02	816/816	NODE_298_length_1372_cov_66.1309	412..1227	Aminoglycoside resistance	V00359					
	strB	100	837/837	NODE_10_length_63444_cov_12.6064	4847..5683	Aminoglycoside resistance	M96392					
	strA	100	804/804	NODE_10_length_63444_cov_12.6064	5683..6486	Aminoglycoside resistance	AF321551					
	blaTEM-1C	100	861/861	NODE_124_length_13854_cov_12.9999	12648..13508	Beta-lactam resistance	FJ560503					
	sul1	100	927/927	NODE_128_length_13170_cov_11.1336	4483..5409	Sulphonamide resistance	CP002151					
	sul2	100	816/816	NODE_10_length_63444_cov_12.6064	6547..7362	Sulphonamide resistance	GQ421466					
	tet(A)	100	1200/1200	NODE_124_length_13854_cov_12.9999	5684..6883	Tetracycline resistance	AJ517790					
	dfrA1	100	474/474	NODE_128_length_13170_cov_11.1336	2708..3181	Trimethoprim resistance	JQ690541					
SS-42	strB	100	837/837	SS-42_NODE_163_length_8528_cov_34.9508	3748..4584	Aminoglycoside resistance	M96392	gyrA p.S83L	TCG → TTG	S → L	Quinolones,Fluoroqui	15848289
	aph(3')-IIa	99.75	795/795	SS-42_NODE_211_length_4777_cov_2.31527	425..1219	Aminoglycoside resistance	X57709					
	strA	99.88	804/804	SS-42_NODE_163_length_8528_cov_34.9508	4584..5387	Aminoglycoside resistance	AF321551					
	catA1	99.76	660/412	SS-42_NODE_807_length_412_cov_0.863158	1..412	Phenicol resistance	V00622					
	sul2	100	816/816	SS-42_NODE_163_length_8528_cov_34.9508	5448..6263	Sulphonamide resistance	GQ421466					
	tet(A)	100	1200/1172	SS-42_NODE_163_length_8528_cov_34.9508	1241..2412	Tetracycline resistance	AJ517790					
	dfrA1	99.79	474/474	SS-42_NODE_39_length_32995_cov_31.767	30062..30535	Trimethoprim resistance	X00926					
SS-43	strB	100	837/837	SS-43_NODE_157_length_8528_cov_61.0765	3620..4456	Aminoglycoside resistance	M96392	gyrA p.S83L	TCG → TTG	S → L	Quinolones,Fluoroqui	15848289
	strA	99.88	804/804	SS-43_NODE_157_length_8528_cov_61.0765	4456..5259	Aminoglycoside resistance	AF321551					
	sul2	100	816/816	SS-43_NODE_157_length_8528_cov_61.0765	5320..6135	Sulphonamide resistance	GQ421466					
	tet(A)	100	1200/1172	SS-43_NODE_157_length_8528_cov_61.0765	1113..2284	Tetracycline resistance	AJ517790					
	dfrA1	99.79	474/474	SS-43_NODE_42_length_32995_cov_32.1312	30062..30535	Trimethoprim resistance	X00926					
